# Impressions That Last: Particularly Negative and Positive Experiences Reported by Parents Five Years after the End of a Child’s Successful Cancer Treatment or Death

**DOI:** 10.1371/journal.pone.0157076

**Published:** 2016-06-07

**Authors:** Lisa Ljungman, Marike Boger, Malin Ander, Brjánn Ljótsson, Martin Cernvall, Louise von Essen, Emma Hovén

**Affiliations:** 1 Clinical Psychology in Healthcare, Department of Public Health and Caring Sciences, Uppsala University, Uppsala, Sweden; 2 Department of Clinical Neuroscience, Division of Psychology, Karolinska Institutet, Stockholm, Sweden; TNO, NETHERLANDS

## Abstract

**Objective:**

To describe the experience of parenting a child diagnosed with cancer by examining particularly negative and positive experiences reported by parents of childhood cancer survivors and parents of children lost to cancer.

**Methods:**

168 parents (88 mothers, 80 fathers) participated. Data were collected five years after the end of successful treatment or the child’s death. The parents’ experiences were identified by open-ended semi-structured questions about particularly negative and positive experiences of the child’s cancer. An inductive approach was used in which the manifest verbal content of the answers was analysed using content analysis.

**Results:**

The analysis revealed eight categories of negative experience (child late effects; distressing events; healthcare; impaired relationships; long-term psychological consequences; own reactions; surrounding institutions; the fact that the child got cancer) and seven categories of positive experience (healthcare; improved relationships; long-term consequences for the child; personal development; support systems; treatment outcome; unexpected joy). The categories were related to past events or to the present situation. The findings indicate variations in experiences between parents of survivors and bereaved parents, and between fathers and mothers, as some experiences were only reported by parents of survivors and some experiences were only reported by mothers.

**Conclusions:**

The results highlight the importance of past and present events to parents, and accordingly the long-lasting impact of paediatric cancer on parents. The results also point to the wide range of negative as well as positive experiences involved in parenting a child diagnosed with cancer, and provide a comprehensive understanding of the overall experience for parents of children with cancer. Specifically, the findings give guidance to healthcare providers by illustrating the need to provide healthcare personnel with continuous training in communication skills, offering parents opportunities to meet other parents in the same situation and increasing the access to psychosocial supportive services and psychological care.

## Introduction

Even though the overall survival rate for childhood cancer has improved dramatically over the past decades [1] childhood cancer still constitutes a serious threat to the child’s life, imposing multiple sources of stress for the affected child and its family [2,3]. Studies of parental psychological stress show that parents of children diagnosed with cancer report high levels of symptoms of depression, anxiety and posttraumatic stress in a short [4,5] as well as a long-term perspective [6–8].

Parenting a child diagnosed with cancer involves numerous challenging events, often over a long time [3]. To reach a comprehensive understanding of this experience it is important to map the events that parents recognise as particularly negative as well as positive. In terms of negative experiences, previous research has reported that parents of children being treated for cancer perceive seeing the child very ill and suffering from treatment side effects [9,10] and supporting the child through painful medical procedures [11] as particularly upsetting. Furthermore, coping with the potential risk that the child might die from the disease has been recognised as highly distressing [8,12]. After the end of the child’s treatment, parents have reported experiencing disturbing memories from the time when the child was ill [13,14], persistent worry [6,11], uncertainty, fear of relapse and relationship strains [2,15]. Studies examining which of these experiences, or possibly other experiences omitted in the previous literature, parents perceive as particularly negative in a long-term perspective are lacking.

In addition to negative experiences, parents of children diagnosed with cancer have reported positive experiences in relation to their child’s cancer, such as changed values and improved relationships [16]. Experiences of such positive psychological development have been conceptualised as posttraumatic growth (PTG) [2,17,18]. The prevalence of PTG has not yet been determined, but previous research indicates that the majority of parents report positive changes as a consequence of the child’s cancer [16]. However, the focus on positive experiences in the previous literature has been limited to positive psychological development rather than positive experiences per se.

Parents’ experiences related to their child’s cancer are inevitably dependent on the final outcome of the treatment; i.e. whether or not the child survives. For bereaved parents the emotional burden is substantial. Bereaved parents report high levels of symptoms of anxiety, depression, posttraumatic stress and complicated grief several years after their child’s death [19–22]. Nevertheless, studies indicate that PTG is also evident in parents of children lost to cancer [23,24].

To the best of our knowledge, no study has explored particularly negative and positive experiences in relation to the child’s cancer as reported by parents of survivors and bereaved parents. The mapping of these experiences is essential to increase the understanding of the situation faced by parents of children diagnosed with cancer. In addition to the important theoretical contribution, this knowledge will provide guidance for healthcare providers and other supportive bodies, which can strive to minimise parents’ negative experiences and support their positive experiences on the basis of these results.

Using a long-term perspective, the aim of the present study was to fill this gap in the literature and add a comprehensive understanding of the experience of parenting a child with cancer. The study design allows for examining the experiences that parents perceive as most significant when they have gone through the child’s disease trajectory, from the time of the diagnosis through the treatment to the period following completion of the treatment and into the long-term survival or death of the child. In order to achieve the study aim, parents of survivors and bereaved parents (five years after the end of successful treatment or five years after the death of the child) were asked to describe particularly negative and positive experiences in relation to their child’s cancer disease.

The following research questions were posed:

1. Do mothers and fathers of childhood cancer survivors report any particularly negative and/or positive experiences in relation to the child’s cancer disease five years after end of the child’s successful treatment, and if so, what are they?
2. Do bereaved mothers and fathers report any particularly negative and/or positive experiences in relation to the child’s cancer disease five years after the child’s death, and if so, what are they?

## Materials and Methods

The current study was conducted and reported in accordance to the Consolidated Criteria for Reporting Qualitative Research (COREQ) checklist [25]. The study is part of a project with the overall aim of investigating psychological and economic consequences of parenting a child diagnosed with cancer. The project includes seven assessments (T1-T7) from one week after the time of diagnosis (T1) up to five years after the end of successful treatment or the child’s death (T7). The end of successful treatment is the time at which the child completed treatment considered successful by the responsible paediatric oncologist. For children who had gone through transplantation it was decided, by discussion with paediatric oncologists, that six months after transplantation should be considered equivalent to the end of successful treatment. The current study was based on data collected at the last assessment point (T7), which were on average collected 2043 days (SD = 74) after the end of successful treatment or the child’s death.

### Sample

Parents of children diagnosed with cancer at four of the six Swedish paediatric oncology centres (Gothenburg, Linköping, Umeå and Uppsala) were consecutively recruited during an 18 month-period from 2002 to 2004. The following eligibility criteria were applied: Swedish and/or English-speaking parents (including step-parents) of children 0–18 years, diagnosed ≤14 days previously with a primary cancer diagnosis and scheduled for chemotherapy and/or radiotherapy. Additionally, parents should have contact with the child, be considered by the responsible paediatric oncologist to be physically and emotionally capable of participating and have access to a telephone. Of 325 eligible parents, 259 (130 mothers; 129 fathers), representing 139 families, consented to participate at T1 (80% response rate). In the present study, to achieve a homogenous sample we chose to exclude the step-parents (n = 1) who participated at T7. The study group thus consists of the 168 parents, representing 105 families, participating at the last assessment (T7). No differences were found between those who participated at T7 (n = 168) and those who did not (n = 91) regarding age, gender, civil status or child diagnosis (CNS tumour versus other diagnoses) at T1. However, non-participants at T7 had a lower educational level (χ^2^ = 11.12, p = 0.004) at T1, and more non-participants than participants at T7 had no other child than the child diagnosed with cancer at T1 (χ^2^ = 8.16, p = 0.017). Table 1 presents characteristics of the participants at T7. A more detailed description of study enrolment and sample at prior assessments (T1-T6) is available in a previous publication [7].

**Table 1:** Characteristics of parents (n = 168) and children (n = 105) five years after the child’s cancer treatment or death.

**Parent characteristic**	**Parents of survivors**	**Bereaved parents**
	**(n = 132)**	**(n = 36)**
Age in years, n(%)		
<40	20(15.2)	11(30.6)
40–49	69(52.3)	16(44.4)
≥50	41(31.1)	6(16.7)
Not stated	2(1.5)	3(8.3)
Gender, n(%)		
Female	68(51.5)	20(55.6)
Male	64(48.5)	16(44.4)
Nationality, n(%)		
Swedish	122(92.4)	30(83.3)
Other	7(5.3)	3(8.3)
Not stated	3(2.3)	3(8.3)
Civil status, n(%)		
Spouses/couples	111(84.5)	31(86.1)
Single	21(15.9)	5(13.9)
Education, n(%)		
≤Nine year elementary	15(11.4)	4(11.1)
Upper secondary	70(53.0)	14(38.9)
University	44(33.3)	15(41.7)
Not stated	3(2.3)	3(8.3)
**Child characteristic**	**Survivors**	**Deceased**
	**(n = 82)**	**(n = 23)**
Age^a^ in years, n(%)		
0–3	24(29.3)	7(30.4)
4–7	23(28.0)	5(21.7)
8–12	20(24.4)	7(30.4)
13–18	15(18.3)	4(17.4)
Gender, n(%)		
Female	38(46.3)	11(47.8)
Male	44(53.7)	12(52.2)
Type of cancer, n(%)		
Leukaemia	31(37.8)	8(34.8)
CNS tumour	8(9.8)	5(21.7)
Other solid tumours	43(52.4)	10(43.5)

^a^ Variable assessed at the time of diagnosis.

### Procedure

Parents who met the inclusion criteria were provided with written and oral information about the study by a coordinating nurse at the respective centre within the first two weeks after the child’s diagnosis. The same nurse asked parents for oral informed consent to participate and permission to be contacted over the telephone by a research assistant. At the end of each interview (T1-T6) oral consent to contact the parent again (T2-T7) was acquired by a research assistant. Oral consent was obtained in accordance with the standards for informed consent for data collection via telephone at the time the study was conducted. Consent was documented by the coordinating nurse at T1 and thereafter by the research assistant who conducted the respective interview. Ethical approval of the study procedure, including the process to obtain consent, was obtained in 2002 from the local research ethics committees at the respective faculties of medicine in Gothenburg (Research Ethics Committee, Faculty of Medicine, University of Gothenburg), Linköping (Research Ethics Committee, Faculty of Medicine, Linköping University), Umeå (Research Ethics Committee, Faculty of Medicine and Odontology, Umeå University) and Uppsala (Research Ethics Committee, Faculty of Medicine, Uppsala University) when the study was launched (DNR:02–006). In 2004 the organisation of ethical vetting in Sweden changed, from being administered by local ethical research committees to being administered by regional ethical committees. The study procedure including the procedure to obtain consent was approved by the Regional Ethical Review Board in Uppsala in 2008 (DNR: 2008/109).

### Interviews

A telephone interview about parents’ particularly positive and negative experiences in relation to their child’s cancer disease was performed by a nurse or a psychologist. All interviewers were female. The procedure of the interviews was as follows: First, the parent was asked the following questions: “Have you had any particularly negative experience in relation to your child’s cancer disease?” and “Have you had any particularly positive experience in relation to your child’s cancer disease?” Parents were asked to answer with one of the following answering alternatives: Yes; No; I am not sure. Parents who answered “Yes” or “I am not sure” regarding negative and/or positive experiences were asked to describe these. The answers were simultaneously transcribed by the interviewer.

### Analysis

The manifest verbal content of the answers about negative and/or positive experiences were analysed with content analysis in accordance with the guidelines from Graneheim and Lundman [26]. Negative and positive experiences were analysed separately. Initially all authors read and reread the transcripts to become familiar with the data. The analysis was conducted in five steps. Answers by fathers of survivors, mothers of survivors, bereaved fathers and bereaved mothers were analysed separately in the first three steps of the analysis. In the first step meaning units were identified and reduced to condensed meaning units by Author 2 (MB). The meaning units and condensed meaning units were reviewed independently by Author 4 (MC) and Author 6 (EH). Disagreements, for example with regard to the level of condensing were discussed until consensus was reached. In the second step Author 1 (LL) and Author 3 (MA) abstracted the condensed meaning units into codes. This step was first conducted independently, and thereafter the codes were compared and disagreements discussed and negotiated. In the third step, the codes were compared based on differences and similarities and categorised into subcategories, first independently by Author 1 (LL) and Author 3 (MA), and then by these two authors working together. Disagreements were discussed until consensus was reached. In the fourth step, all identified subcategories for the four subgroups (fathers of survivors, mothers of survivors, bereaved fathers and bereaved mothers) were reviewed by Author 1 (LL), Author 2 (MB), Author 3 (MA) and Author 6 (EH), resulting in a new set of mutually exclusive subcategories. The fifth step was conducted by Author 1 (LL), Author 2 (MB), Author 3 (MA) and Author 6 (EH) who analysed and organised subcategories into mutually exclusive categories. When the categories emerged it was apparent that their content was related to either the past or the present, and the categories were therefore classified within these two overarching themes. Finally Author 1 (LL) and Author 3 (MA) re-read all transcripts, through each meaning unit, condensed meaning unit, code, subcategory and category, to check the agreement of the data. The few disagreements found between Author 1 (LL) and Author 3 (MA) were resolved by re-coding a few codes and re-sorting these into another subcategory until consensus was reached among all authors. The analytical procedure, including the initial steps of independent coding and the complementary confirmatory analyses, was applied to increase the trustworthiness of the analysis [27]. To further increase the credibility of the results, a parent of a child who had completed treatment for cancer approximately five years previously carefully read the results of the analyses and reflected upon these together with the authors.

## Results

Particularly negative experiences in relation to the child’s disease were reported by 63% of fathers and 69% of mothers of survivors (Table 2). Among bereaved parents, 81% of fathers and 75% of mothers reported having had a particularly negative experience. Particularly positive experiences were reported by 92% of fathers and 90% of mothers of survivors. The corresponding figures for positive experiences in bereaved parents were 63% for fathers and 90% for mothers.

**Table 2:** Parents’ answer to the questions “Have you had any particularly negative/positive experience in relation to your child’s cancer disease?” five years after the child’s cancer treatment or death.

	Parents of survivors			Bereaved parents		
	n(%)			n(%)		
	Fathers	Mothers	Total	Fathers	Mothers	Total
	(n = 64)	(n = 68)	(n = 132)	(n = 16)	(n = 20)	(n = 36)
Negative experience/s						
Yes	40(62.5)	47(69.1)	87(65.1)	13(81.3)	15(75.0)	28(77.8)
Don’t know	1(1.6)	2(2.9)	3(2.3)	1(6.3)	1(5.0)	2(5.6)
No	23(35.9)	19(27.9)	42(31.8)	2(12.5)	4(20.0)	6(16.7)
Positive experience/s						
Yes	59(92.2)	61(89.7)	120(90.1)	10(62.5)	18(90.0)	28(77.8)
Don’t know	2(3.1)	4(5.9)	6(4.5)	0(0)	1(5.0)	1(2.8)
No	3(4.7)	3(4.4)	6(4.5)	6(37.5)	1(5.0)	7(27.8)

The content analysis resulted in eight categories of negative experience and seven categories of positive experience (Table 3 and Table 4). In total, 22 subcategories were identified; nine for negative experiences and 13 for positive experiences. The categories were classified into two overarching themes; Past and Present. The text of the results, which aims to illustrate the categories and where applicable subcategories, includes quotations. Unless otherwise stated, the categories and subcategories were represented by parents in all subgroups; i.e. fathers and mothers of survivors and bereaved fathers and mothers (Table 3 and Table 4).

**Table 3:** The categories and subcategories for negative experiences in parents of children with cancer and whether the category/subcategory was represented in parent subgroups (fathers and mothers of survivors, and bereaved fathers and mothers). Subcategories and categories are presented in alphabetical order.

Theme	Category	Subcategory
Past	Distressing events	
		*Acute medical conditions*^a^^,^^b^
		*Receive negative information*^b^
		*See the child suffer*^a^
		*Waiting*^a^
	Healthcare	
		*Approach from healthcare personnel*
		*Care outside the paediatric oncology centre*^a^
		*Concerns not taken seriously*
		*Lack of psychological care*^a^
		*Mistakes and carelessness*
	Own reactions^a^^,^^b^	*N/A*
	Surrounding institutions^a^	*N/A*
	The fact that the child got cancer	*N/A*
Present	Impaired relationships^b^	*N/A*
	Child late effects^a^^,^^b^	*N/A*
	Long-term psychological consequences	*N/A*

^a^ Not represented in the subgroup of bereaved fathers.

^b^ Not represented in the subgroup of bereaved mothers.

**Table 4:** The categories and subcategories for positive experiences in parents of children with cancer and whether the category/subcategory was represented in parent subgroups (fathers and mothers of survivors, and bereaved fathers and mothers). Subcategories and categories are presented in alphabetical order.

Theme	Category	Subcategory
Past	Healthcare	
		*Healthcare personnel*
		*Healthcare system*^a^
		*Play therapy and hospital clowns*
	Support systems	
		*Social network*
		*Societal bodies*^a^
	Treatment outcome^a^^,^^b^	*N/A*
	Unexpected joy^b^	
		*A happy child*^b^
		*Joyful moments*^b^^,^ ^c^
		*Time with the family*^a^^,^^b^
Present	Improved relationships	
		*Deepened relationships*
		*New relationships*
	Long-term consequences for the child^a^^,^^b^	*N/A*
	Personal development	
		*Changed self-perception*
		*New view of life*
		*Knowledge and skills*^a^^,^^b^

^a^ Not represented in the subgroup of bereaved mothers.

^b^ Not represented in the subgroup of bereaved fathers.

^c^ Not represented in the subgroup of fathers of survivors.

### Past negative experiences

#### Distressing events

Answers in the category **Distressing events** concern specific events during the time of the child’s illness and treatment which parents had perceived as particularly upsetting. The category includes the subcategories: ***Acute medical conditions***; ***Receive negative information***; ***See the child suffer*** and ***Waiting***.

***Acute medical conditions*** concerns occasions on which the child became very ill, such as having seizures. Parents described these experiences as horrifying, and several mentioned this being related to fear that the child was going to die. A mother of a survivor described this experience: *“After two treatments he got so terribly ill and I thought he would die*. *It’s nobody’s fault*, *that’s just the way the treatment worked on him*, *but he got so so sick*…*”*. Experiences in this subcategory were only reported by parents of survivors.

***Receive negative information*,** reported by all subgroups except bereaved mothers, involves descriptions of the period when parents were first told that the child had cancer, but also other occasions when they received negative information, such as information on the progression of the disease. Fathers who had lost their child to cancer mentioned the specific moment when the doctors informed them that the child was going to die. A bereaved father said: *“When the doctors said that there was no hope*, *and when they told her that she was going to die… That was the worst of the worst”*.

***See the child suffer***, reported by all subgroups except bereaved fathers, includes descriptions of the suffering that the child had been put through during treatment, such as painful medical procedures, and when the child suffered from side effects of the treatment. Parents mentioned having had to physically force the child through procedures which had been very upsetting both to the child and to the parents. A mother of a survivor said: *“When they were puncturing her back… we were several people who held her down*, *and she just screamed–‘Mummy*, *save me*!*’”*

***Waiting*** was reported by all subgroups except bereaved fathers, and involves answers regarding waiting, for example before receiving the diagnosis, for information on disease progress and for transplantation. A mother of a survivor said: *“Waiting for various answers is incredible demanding*. *You are so vulnerable”*.

#### Healthcare

The category **Healthcare** includes five subcategories: ***Approach from healthcare personnel***; ***Care outside the paediatric oncology centre***; ***Concerns not taken seriously***; ***Lack of psychological care*** and ***Mistakes and carelessness***.

***Approach from healthcare personnel*** includes answers about healthcare personnel being unfriendly, stressed or having approached the sick child in a disrespectful way. Several parents mentioned situations in which the personnel had been stressed and/or insensitive during medical procedures which had frightened the child, causing unnecessary distress. Bereaved parents mentioned that personnel had talked about death in an insensitive manner in front of the child. A bereaved mother said: *“When he [the doctor] said to me*, *out loud in front of the child*: *‘You have to accept that there is nothing more to do*, *that she will die*!*’ He [the doctor] could have asked me to follow him out”*.

***Care outside the paediatric oncology centre*,** reported by all subgroups except for bereaved fathers, includes descriptions of negative experiences at local hospitals and adult oncology centres. Parents attributed these to lack of specific paediatric cancer competence, less engaged staff and a higher degree of responsibility for the medical care being put on the parents when outside the paediatric oncology centre. A mother of a survivor said: *“She [the paediatric oncologist] sent our papers to the Sahlgrenska [adult oncology centre] so that we wouldn’t have to inform them about everything*, *but the doctor there [at the adult oncology centre] still didn’t know anything”*.

***Concerns not taken seriously*** includes answers about not having been taken seriously when first contacting the healthcare for the child’s symptoms, i.e., prior to having received the diagnosis. Parents described having had to fight in order to get the correct diagnosis, which had negatively affected their trust in the healthcare system. Among bereaved parents there were questions about whether not having been taken seriously was related to the child’s death. A bereaved mother said: *“They didn’t listen to us when she had a fever… It is something that you will never get over*, *that we might still have had her if she had got the antibiotics”*.

***Lack of psychological care*** was mentioned by all subgroups except bereaved fathers, and encompasses answers about a lack of psychologists and counsellors at the paediatric oncology centre. A mother of a survivor said: *“The fact that there was no counsellor or psychologist available*. *That should be provided when they deliver such information [about the cancer diagnosis]*. *You don't think about that then*, *but now*, *afterwards”*. Some bereaved mothers mentioned not having been offered any psychological care after the child’s death.

***Mistakes and carelessness*** includes answers about occasions when the child became very sick due to medical misjudgements, when medical exams included in the treatment protocol were omitted or when doctors made errors in diagnostics. Bereaved parents expressed concerns about whether or not the child could have been saved if mistakes had not been made. A bereaved father said: *“He was under examination and they concluded that something was wrong*, *but they didn’t inform us about this at an early stage*. *Had they told us this*, *then maybe he would still have been alive”*.

#### Own reactions

**Own reactions** includes descriptions of the parents’ own strong negative reactions during the treatment period, such as being in shock, feeling angry and being worried and afraid about whether or not the child would survive. A father of a survivor said: *“The fear when she got ill*, *before we knew that it would end well”*. Parents described having striven to put these reactions aside as they wanted to stay strong and focus all their energy on their child. Some parents reported having neglected their own medical health concerns during the child’s treatment. This category was only mentioned by parents of survivors.

#### Surrounding institutions

***Surrounding institutions*** includes answers about private insurance companies as well as the national healthcare insurance agency. Parents described having received very little financial support from insurance companies, having had contact with personnel perceived as rude and having had problems getting approval for paid sick leave. A father of a survivor said: *“The worst thing was the national healthcare insurance agency*. *They were so determined to get people to work*. *Had no empathy at all*. *It would have been good for them to try it themselves… It’s the last thing you need when you are in that situation”*. Some mentioned a lack of support from their employer and negative experiences from the child’s school; for example, the school failing to adapt to the child’s needs. All subgroups except bereaved fathers mentioned issues included in this category.

#### The fact that the child got cancer

Parents in all subgroups mentioned the fact that their child got cancer and everything that comes with this as a particularly negative experience, such as having to go through the treatment. Bereaved parents mentioned the overall situation; that the child got cancer and eventually died from the disease.

### Present negative experiences

#### Impaired relationships

**Impaired relationships** was mentioned by all subgroups except bereaved mothers and involves answers about relationships that had been put under strain due to the disease, which had resulted in impaired relationships. Parents mentioned impaired relationships within the family, often with the siblings of the sick child as well as with friends. A mother of a survivor said: *“The friends who have disappeared*, *we weren’t able to maintain contact and nor did they”*.

#### Child late effects

This category, reported by parents of survivors, includes answers about current physical and psychological late effects such as persistent pain, neurological deficits, possible infertility, cognitive impairments, social difficulties and psychological ill-health. A father said: *“His situation with friends*, *the way it turned out afterwards*. *He has been so alone*. *It could be his personality of course*, *but he has missed so much that his peers have done*, *and at the same time he is so mature”*.

#### Long-term psychological consequences

All subgroups described negative long-term psychological consequences such as intense anxiety when reminded of the child’s disease. Parents also mentioned having a persistent worry related to the child’s present and possible future late effects, and fear of relapse. Furthermore, parents described depressive feelings that had arisen after the end of successful treatment, and that the negative emotions that were “put aside” during the child’s disease period had become more intense after the end of treatment. Parents described how they were more sensitive to stress than before the child got ill, and that the child’s disease had led to perceiving the world as an unsafe place. A mother of a survivor said: *“It is so close to the surface*. *In my eyes he will never be completely cured*. *I have to control myself not to pass my anxiety onto him*, *for example if he gets a cold”*.

### Past positive experiences

#### Healthcare

Positive experiences related to healthcare were categorised as: ***Healthcare personnel***; ***Healthcare system*** and ***Play therapy and hospital clowns***.

***Healthcare personnel*** includes descriptions of the personnel as having been supportive, genuine and warm, showing that they truly cared. The importance of a positive and cheerful approach was highlighted. Bereaved parents also mentioned personnel who had shown genuine grief and sorrow in relation to the death of the child. A bereaved mother said: *“The fantastic personnel that we met*… *They did so much for us*, *wonderful care*. *They made us laugh despite our situation*. *I still meet them sometimes in the city*, *it’s a warm feeling*, *kind hugs*. *I feel like they are grieving with us”*.

***Healthcare system*** includes experiences of the Swedish healthcare system as functioning well. Parents mentioned that the care at the paediatric oncology centre had been excellent, trustworthy, professional and competent. A mother of a survivor said: *“That the healthcare system worked so well*. *We felt secure about the care he got”*. Some parents of survivors mentioned the importance of the continuous support they had received from the paediatric oncology centre after the end of treatment. Among bereaved parents, only fathers gave answers in this subcategory.

***Play therapy and hospital clowns*** were described as highly appreciated supportive services. Parents stated that these had helped them put up with the struggle by cheering them up, and by giving them important opportunities to socialise with parents in similar situations. The play therapy and hospital clowns were described as positive for the whole family, and an important complement to the regular care. A mother of a survivor said: *“It [the play therapy] has been a survival strategy for the kids*, *the siblings and us parents*. *That’s where resources should be allocated*. *It’s worth more than gold”*.

#### Support systems

Positive experiences of support systems includes the subcategories: ***Social network*** and ***Societal bodies***.

***Social network*** includes answers about family and friends that had ‘been there’. Parents described how friends and relatives had truly cared, and that, for example, neighbours had put extra effort into helping by raising money for the family to do something fun. Parents also mentioned the importance of support from other parents of children diagnosed with cancer. A father of a survivor said: *“Your social network was there and it worked well*. *You got confirmation of that at the time”*.

Positive experiences categorised as ***Societal bodies*** concern organisations such as the Swedish Childhood Cancer Foundation and the Ronald McDonald House which had enabled the families to do things such as travelling together or living with the sick child during hospital stays. Furthermore, private insurance companies as well as the national insurance agency were mentioned. Some stated that the insurance system had been proven to work smoothly, which resulted in the parents being economically well supported during the time when they were not able to work due to the child’s condition. No bereaved mother gave an answer in this subcategory.

#### Treatment outcome

Only parents of survivors mentioned answers regarding treatment outcome; e.g. answers about the successful treatment outcome ‒ that the child was cured, and gratitude as a result of this. A father of a survivor said: *“That it went well*, *that was crucial*, *otherwise the feelings would have been different”*.

#### Unexpected joy

Unexpected joy was mentioned by all subgroups but bereaved fathers. The category includes the subcategories: ***A happy child***; ***Joyful moments*** and ***Time with the family***.

***A happy child*** includes answers regarding the child having been positive, brave and happy during treatment. Parents mentioned that the child liked to be at the hospital and even looked forward to going there. Some related the wellbeing of the child to the fantastic job that healthcare personnel had done. A mother of a survivor said: *“She has always been so happy when we have gone there [the hospital]*, *even though it has been tough”*.

***Joyful moments*** were mentioned only by mothers. The subcategory includes statements about happy moments during the treatment, such as eating ice cream in the hospital park with the sick child, or when a Christmas dinner was arranged at the clinic. These moments of joy were described as being more intense than normally. A mother of a survivor said: *“It’s negative that the child got cancer*, *but included in that there are so many moments of enormous joy*. *For example*, *when he could eat breakfast*, *then I could cry of joy*. *We held a party every time he came home and could eat”*.

***Time with the family*** was only mentioned by parents of survivors. This subcategory involves answers such as having shared a lot of experiences with the family, and having got the chance to really talk to the child during the time spent at the hospital. A father of a survivor said: *“That we got more time with her*, *could talk to her; it was enriching and positive in the middle of the misery”*.

### Present positive experiences

#### Improved relationships

**Improved relationships** consists of two subcategories: ***Deepened relationships*** and ***New relationships***.

***Deepened relationships*** mainly includes answers about the child’s disease as having brought the family closer together. Parents mentioned talking more openly in the family, being closer to the surviving child and a strengthened marital relationship. A father of a survivor said: *“It has strengthened the whole family; we have a stronger bond and connection*. *We enjoy being with each other and being together”*. Parents mentioned that the experience had enriched relationships with other relatives and that relationships with old friends had become more open and honest.

***New relationships*** includes having met people through the child’s disease; both parents and other children that had become friends. Some parents mentioned families they had met through their child’s disease which had become friends for life. Parents described that they had a special relationship with these new friends, and that they shared thoughts and feelings with them that they could not do with other friends.

#### Long-term consequences for the child

Descriptions of positive long-term consequences for the child were mentioned by parents of survivors; for example that the child had matured with the cancer experience and had developed his/her ability to see things from different perspectives. Some mentioned that the child had become more empathetic, no longer focusing on minor problems. A father said: *“The child has matured*, *kind of*, *can keep up a discussion and has empathy*. *Doesn’t whine about small problems”*.

#### Personal development

Experiences related to personal development were reported by all subgroups and were categorised as: ***Changed self-perception***; ***New view of life*** and ***Knowledge and skills***.

***Changed self-perception*** involves perceiving oneself as stronger and more independent after the child’s disease. Parents attributed this to having proven to themselves that they could cope with more than they thought. Parents also mentioned that they saw themselves as more humble and empathetic after the child’s disease, and that they wanted to use the experience to help others in difficult situations. A father of a survivor said: *“Got to know myself in a completely different way*, *and I have greater understanding of other people”*.

***New view of life*** involved perceiving the cancer experience as a wake-up call, making parents aware of what is truly important in life; often involving the importance of the family in general, and the children in particular. Parents described a wish not to “postpone life until tomorrow”, and an increased appreciation of everyday life. A bereaved mother said: *“I have learned to appreciate life in a different way*. *I actually live; I don’t just exist*.*”*

Parents of survivors mentioned ***Knowledge and skills***, for example including how to manage the healthcare system and skills such as how to talk to doctors and healthcare personnel. Parents also mentioned having gained information about diseases in general and childhood cancer in particular, including the fact that cancer is not necessarily fatal. A father of a survivor said: *“Learned a lot about cancer*, *that it is curable*. *That has taken the edge off cancer*. *It isn’t that horrifying”*.

## Discussion

To reach a comprehensive understanding of the experience of parenting a child diagnosed with cancer, we examined particularly negative and positive experiences reported by parents of survivors and by bereaved parents five years after the end of successful treatment or the child’s death. The results show that negative and positive experiences were commonly reported. Negative experiences were reported by 65% of parents of survivors and by 78% of bereaved parents. The corresponding figures for positive experiences were 90% for parents of survivors, and 78% for bereaved parents. The content analyses revealed eight categories of negative experience and seven categories of positive experience which were related to past events or to the present situation. The results points to the wide range of experiences involved in parenting a child with cancer, and to the long-lasting impact of childhood cancer on parents.

Negative experiences of the past concerned distressing events, healthcare, the parents’ own reactions, surrounding institutions and the fact that the child got cancer. Of interest is that negative experiences of healthcare personnel were always related to a certain nurse/doctor, never to the whole team. In contrast, the whole team was often mentioned in positive terms. These findings can be interpreted as indicating that negative experiences of healthcare personnel are isolated to certain individuals, and contrast with the overall positive impressions of healthcare personnel. However, the result underscores the importance of continuous training of healthcare personnel in communication skills, as well as supervision and support of personnel with regard to how to approach parents and children with cancer. Healthcare personnel should bear in mind that every single encounter with the child and family matters, and that one single negative interaction can result in a negative experience that parents may carry with them for many years. Furthermore, parents described an overall lack of psychological care services in the paediatric oncology centres, including a lack of opportunities to meet counsellors and psychologists. This points to room for improvement with regard to access to psychological care, both during the time of diagnosis and treatment as well as after the end of successful treatment or in the aftermath of the child’s death. Bereaved parents also mentioned concerns about whether mistakes and carelessness had led to the child’s death. This underscores the importance of examining whether bereaved parents experience such concerns. Previous research has pointed to the potential benefits of closing sessions with parents following the death of a child [28]. Including opportunities during these sessions for parents to express concerns regarding potential mistakes would enable possible misunderstandings to be cleared up.

Long-term psychological consequences, still present five years after the end of successful treatment or the child’s death, was mentioned by all subgroups. The category includes aspects such as anxiety at check-ups, anxiety and worry when the child developed routine illnesses and fear of recurrence. These experiences resemble symptoms of traumatic stress [29,30] which have been reported by parents of children diagnosed with cancer [7,8,14]. Our results highlight the relevance of such experiences in a long-term perspective for both fathers and mothers of survivors and for bereaved fathers and mothers.

The high percentage of parents reporting positive experiences should be highlighted. The literature on parents’ experiences related to a child’s cancer disease almost exclusively concern negative experiences other than PTG [17,18]. Our results show that parents of children diagnosed with cancer experience numerous positive experiences which are not necessarily captured by the PTG construct, such as positive interactions with the healthcare personnel, joyful experiences during the child’s treatment and positive long-term development of the child. A comprehensive description of the experience of parenting a child diagnosed with cancer should thus include not only negative but also broadly defined positive experiences.

The positive experiences related to the past concern the healthcare, support systems, treatment outcome and unexpected joy. All subgroups mentioned positive experiences related to healthcare personnel, such as personnel really listening, taking the time the parents needed and showing genuine compassion. Bereaved parents brought up the importance of personnel showing their own emotions, such as grief, in the difficult time of the child’s death. This highlights what has been reported in the literature regarding healthcare personnel behaviour; that honesty and authenticity are appreciated [31,32]. Furthermore, all subgroups brought up positive experiences related to psychosocial supportive services at the hospital such as play therapy and hospital clowns. By making these services available to all families of children diagnosed with cancer, important improvements in psychosocial support could be accomplished.

Positive experiences related to the present situation concerned improved relationships, the child’s long-term development and the parents’ own personal development. Improved relationships and personal development have been reported for this population [16], and could be conceptualised as PTG. Our results thus support previous results on PTG in parents of children diagnosed with cancer [16,23], and show the generalisability of these findings to fathers of survivors and bereaved mothers and fathers, who have often been excluded and/or less represented in the previous literature. The results also indicate that PTG may last over a long period of time. In addition to deepened relationships, which have been described in the previous literature [33], parents described how they had established new friendships through the child’s cancer disease. The potential importance of such relationships in this population should be acknowledged. To enable the development of such new relationships, the healthcare system could prioritise providing parents with opportunities to meet other parents to share experiences, both during and after the child’s treatment or following bereavement.

Compared to parents of survivors, bereaved parents did not report experiences related to acute medical conditions or their own negative reactions during the child’s treatment. It can be hypothesised that in the case of losing one’s child other distressing events such as acute medical conditions become relatively less significant. The same hypothesis applies to the parents’ own reactions; the pain of losing the child outweighs the fear of this event experienced before it actually happened. Additionally, bereaved parents did not report positive experiences related to time spent with the family. It is likely that time spent with the family during the treatment period was far more distressing for families if the child eventually dies which could explain why such experiences during the treatment period were not reported. Also, no bereaved parents reported new knowledge or skills. This subcategory involved answers relating to learning how to manage a cancer disease and having realised that cancer is a curable disease. The absence of such knowledge and skills in bereaved parents is to be expected.

Among bereaved parents, only mothers reported seeing the child suffer and waiting as distressing events. This finding could be discussed in relation to traditional gender roles where the mother acts as the primary caregiver [34]. Accordingly, mothers will likely be more exposed to distressing events such as seeing the child suffer and waiting for information at the hospital. In line with this, no bereaved fathers reported unexpected joy such as seeing the child happy, or having spent time with the family during the treatment, and no father at all reported positive experiences of joyful moments. These findings could also reflect traditional parenting roles as described above; if the fathers participated less in the day-to-day care of the sick child, not only will they have been less exposed to the distressing events related to the child’s treatment, they will also have had fewer opportunities to experience potentially positive events during this time. Among bereaved parents, only fathers reported impaired relationships. This agrees with previous research showing that fathers experience social isolation following the death of the child and that fathers isolate themselves from relationships [35]. Further research is needed to validate these findings on gender differences in the context of parenting a child with cancer, and conclusions with regard to the mechanisms involved await more research. Furthermore, future research should focus on bereaved fathers in order to explore their long-term adaptation and possible need for extra support.

Several of the categories of negative and positive experiences concerned similar topics, such as healthcare, surrounding institutions/support systems, impaired/improved relationships, late effects/long-term psychological consequences for the child and long-term psychological consequences/personal development for the parents themselves. In addition to these, there were descriptions of distressing events as well as unexpected joy during the time of the child’s illness. As a whole, the categories identified can be assumed to represent the most significant experiences for parents of children diagnosed with cancer in Sweden, and specific circumstances determine whether the experiences will be perceived as negative and/or positive. The specific circumstances that turn e.g., healthcare situations into being perceived as negative or positive should be examined further in future research. Such knowledge can be used in clinical care to facilitate positive experiences and reduce or prevent negative ones.

The strengths of the present study include the multicentre approach, the use of a large sample consisting of mothers and fathers of survivors as well as of deceased children and the structured analytic method employed. Furthermore, the long-term follow-up period allows for examination of the experiences perceived by parents as particularly negative or positive over the child’s full disease trajectory. Previous research has indicated that emotionally arousing experiences, both pleasant and unpleasant, are stored longer and more vividly in the memory [36,37], and retrospectively recalling positive and negative experiences can be very informative as to which experiences are particularly significant for the parents. However, some study limitations need to be considered. First, the interviews were simultaneously transcribed instead of recorded and transcribed verbatim. Considering the rather straightforward questions asked, the simultaneous transcription procedure was considered to be an appropriate method. Still, even if the core content of each participant’s answers was captured by this procedure, there might be nuances in the answers that have been overlooked. Secondly, as part of a project investigating psychological and economic consequences for parents, the interviews covered other matters that were not included in the focus of the present study. Covering too many questions and topics is associated with the risk of respondents becoming fatigued or less engaged, which could result in less complete answers. This issue was taken into account *a priori* when the interview was planned, with the aim of finding a proper balance of questions still providing an adequate multi-dimensional assessment that could be completed in a reasonable amount of time. Moreover, it is important to bear in mind that the results should be understood in the context of being a parent of a long-term survivor or several years following bereavement. Future research is encouraged to further examine the experiences captured by the categories/subcategories generated herein to determine their relevance to mothers and fathers of survivors and of deceased children.

## Conclusions

The study explored particularly negative and positive experiences reported by parents of survivors of childhood cancer and bereaved parents in response to semi-structured open-ended interview questions. The results point to the importance of past as well as present events to parents, and accordingly, to the long-lasting impact of parenting a child diagnosed with cancer. With the goal of minimising parents’ negative experiences and facilitating parents’ positive experiences, the results give important clinical guidance such as providing healthcare personnel with continuous training in communication skills, providing parents with opportunities for open discussions with healthcare personnel of aspects such as mistakes in the care of the child, acknowledging the parents’ need to express concerns, offering parents opportunities to meet other parents in the same situation and providing services such as play therapy and hospital clowns. Parents reported limited opportunities for meeting counsellors and psychologists, which suggests that increased access to psychological care should be provided for parents of children diagnosed with cancer. The parents furthermore mentioned a broad variety of positive experiences, illustrating that parenting a child with cancer is a multifaceted experience that includes not only distressing events but also many potential joyful experiences. A comprehensive description of the overall experience of parenting a child diagnosed with cancer should thus include not only negative but also positive experiences. Lastly, the findings indicate variations in experiences between parents of survivors and bereaved parents, and between fathers and mothers. Future studies are encouraged to examine these findings further.
